# Comparing Lung Cancer Screening Strategies in a Nationally Representative US Population Using Transportability Methods for the National Lung Cancer Screening Trial

**DOI:** 10.1001/jamanetworkopen.2023.46295

**Published:** 2024-01-30

**Authors:** Sarah E. Robertson, Nina R. Joyce, Jon A. Steingrimsson, Elizabeth A. Stuart, Denise R. Aberle, Constantine A. Gatsonis, Issa J. Dahabreh

**Affiliations:** 1 CAUSALab, Harvard T.H. Chan School of Public Health, Boston, Massachusetts; 2 Department of Epidemiology, Harvard T.H. Chan School of Public Health, Boston, Massachusetts; 3 Department of Epidemiology, Brown University School of Public Health, Providence, Rhode Island; 4 Department of Biostatistics, Brown University School of Public Health, Providence, Rhode Island; 5 Departments of Mental Health, Biostatistics, and Health Policy and Management, Johns Hopkins Bloomberg School of Public Health, Baltimore, Maryland; 6 Medical & Imaging Informatics Group, Department of Radiological Sciences, David Geffen School of Medicine, University of California, Los Angeles; 7 Department of Biostatistics, Harvard T.H. Chan School of Public Health, Boston, Massachusetts

## Abstract

**Importance:**

The National Lung Screening Trial (NLST) found that screening for lung cancer with low-dose computed tomography (CT) reduced lung cancer–specific and all-cause mortality compared with chest radiography. It is uncertain whether these results apply to a nationally representative target population.

**Objective:**

To extend inferences about the effects of lung cancer screening strategies from the NLST to a nationally representative target population of NLST-eligible US adults.

**Design, Setting, and Participants:**

This comparative effectiveness study included NLST data from US adults at 33 participating centers enrolled between August 2002 and April 2004 with follow-up through 2009 along with National Health Interview Survey (NHIS) cross-sectional household interview survey data from 2010. Eligible participants were adults aged 55 to 74 years, and were current or former smokers with at least 30 pack-years of smoking (former smokers were required to have quit within the last 15 years). Transportability analyses combined baseline covariate, treatment, and outcome data from the NLST with covariate data from the NHIS and reweighted the trial data to the target population. Data were analyzed from March 2020 to May 2023.

**Interventions:**

Low-dose CT or chest radiography screening with a screening assessment at baseline, then yearly for 2 more years.

**Main Outcomes and Measures:**

For the outcomes of lung-cancer specific and all-cause death, mortality rates, rate differences, and ratios were calculated at a median (25th percentile and 75th percentile) follow-up of 5.5 (5.2-5.9) years for lung cancer–specific mortality and 6.5 (6.1-6.9) years for all-cause mortality.

**Results:**

The transportability analysis included 51 274 NLST participants and 685 NHIS participants representing the target population (of approximately 5 700 000 individuals after survey-weighting). Compared with the target population, NLST participants were younger (median [25th percentile and 75th percentile] age, 60 [57 to 65] years vs 63 [58 to 67] years), had fewer comorbidities (eg, heart disease, 6551 of 51 274 [12.8%] vs 1 025 951 of 5 739 532 [17.9%]), and were more educated (bachelor’s degree or higher, 16 349 of 51 274 [31.9%] vs 859 812 of 5 739 532 [15.0%]). In the target population, for lung cancer–specific mortality, the estimated relative rate reduction was 18% (95% CI, 1% to 33%) and the estimated absolute rate reduction with low-dose CT vs chest radiography was 71 deaths per 100 000 person-years (95% CI, 4 to 138 deaths per 100 000 person-years); for all-cause mortality the estimated relative rate reduction was 6% (95% CI, −2% to 12%). In the NLST, for lung cancer–specific mortality, the estimated relative rate reduction was 21% (95% CI, 9% to 32%) and the estimated absolute rate reduction was 67 deaths per 100 000 person-years (95% CI, 27 to 106 deaths per 100 000 person-years); for all-cause mortality, the estimated relative rate reduction was 7% (95% CI, 0% to 12%).

**Conclusions and Relevance:**

Estimates of the comparative effectiveness of low-dose CT screening compared with chest radiography in a nationally representative target population were similar to those from unweighted NLST analyses, particularly on the relative scale. Increased uncertainty around effect estimates for the target population reflects large differences in the observed characteristics of trial participants and the target population.

## Key Points

**Question:**

What is the comparative effectiveness of low-dose computed tomography (CT) vs chest radiography screening strategies evaluated in the National Lung Screening Trial (NLST) in a nationally representative target population of US adults who meet the NLST eligibility criteria?

**Findings:**

In this comparative effectiveness study, transportability analysis was used to reweight NLST data to resemble a nationally representative target population. Estimates of the comparative effectiveness of low-dose CT screening compared with chest radiography on lung cancer–specific and all-cause mortality in the target population of 5.7 million adults were similar to estimates from unweighted NLST analyses, but differences in baseline characteristics between the NLST and the target population resulted in increased uncertainty.

**Meaning:**

These results suggest that low-dose CT screening resulted in improved outcomes compared with chest radiography in a nationally representative population NLST-eligible individuals, indicating that the trial findings are transportable to this target population.

## Teaser

This comparative effectiveness study conducts a transportability analysis of the National Lung Screening Trial to compare effectiveness of low-dose computed tomography and chest radiography screening for US adults.

## Introduction

Lung cancer is the most common cause of cancer death, accounting for an estimated 21.4% of all cancer deaths in 2022.^1^ The American Cancer Society estimated that 236 740 new cases of lung cancer will occur in 2022.^2^ Among lung cancer patients, prognosis correlates with stage at diagnosis, with a 5-year relative survival rate of 59% for patients with local disease compared with 6% for patients with distant disease.^2^ The association between stage at diagnosis and prognosis motivates the investigation of screening with computerized tomography (CT) as a way to detect disease early and allow treatment with curative intent.

The National Lung Screening Trial (NLST), the largest lung cancer screening trial in a high-risk population to date, compared screening with low-dose CT vs single-view posteroanterior chest radiography.^3,4^ The NLST found that screening with low-dose CT reduced lung cancer–specific and all-cause mortality.^3^ In light of these findings, the US Preventive Services Task Force (USPSTF) in 2013 recommended low-dose CT screening for adults 55 to 80 years old who have a 30 pack-year smoking history and currently smoke or have quit within the past 15 years (a grade B recommendation of high certainty of moderate net benefit).^5^ The updated 2021 USPSTF recommendations retained the evidence grade, but lowered the recommended screening cutoffs to 50 years of age and 20 pack-years.^6^

However, the latest USPSTF recommendations have cautioned that the relevance of the NLST findings to populations seen in practice is uncertain, and suggested that research is needed to evaluate whether the effectiveness of CT screening in more diverse community settings differs from that estimated in randomized trials.^6^ Furthermore, the evidence review that informed the latest recommendation stated that “the general US population eligible for lung cancer screening may be less likely to benefit from early detection compared with NLST […] participants because they face a high risk of death from competing causes, such as heart disease and stroke.”^7^ These concerns are important because in 2 meta-analyses from 2021^7,8^ (including the one directly informing the USPSTF recommendations^7^), the NLST contributed 94% of all participants in US-based trials. Several studies have found that NLST participants are younger, have fewer comorbidities, and are more educated compared with populations seen in practice who would be candidates for CT screening.^9,10,11^ Therefore, we need to extend inferences about the comparative effectiveness of screening by low-dose CT vs chest radiography from the NLST to target populations that are representative of populations seen in practice.^12^

Transportability analyses offer an approach for extending inferences from a trial to a nationally representative target population using standardization (eg, via weighting the trial data) to account for differences in the distribution of baseline characteristics between the 2 populations.^13^ In this comparative effectiveness study, we report the results of transportability analyses that combined information from the NLST and the National Health Interview Survey (NHIS)^14^ to answer the question: What would be the comparative effectiveness on lung cancer–specific and all-cause mortality of the low-dose CT vs chest radiography screening strategies evaluated in the NLST, were these strategies to be implemented in a nationally representative target population of NLST-eligible U.S. adults?

## Methods

The institutional review board of Harvard T.H. Chan School of Public Health determined that this study was not human participants research and so did not require review or informed consent. When relevant to transportability analyses, we followed the International Society for Pharmacoeconomics and Outcomes Research (ISPOR) reporting guidelines for comparative effectiveness studies.

### Data Sources and Study Design

#### Randomized Trial

The NLST^3,4,15^ enrolled participants at 33 US centers from August 2002 through April 2004. Eligible individuals were ages 55 to 74 years and current or former smokers with at least 30 pack-years of smoking; former smokers were required to have quit within 15 years of enrollment. Individuals were excluded if they had medical or psychiatric conditions precluding informed consent, had received a low-dose CT chest scan in the past 18 months, had a history of lung cancer or history of nonlung cancer within the past 5 years, had recent medical problems, participated in other studies of cancer screening, had any portion of their lungs removed, were unable to lie flat with arms raised overhead, required home oxygen supplementation, or had metallic implants in the chest or back. Trial participants were randomized to low-dose CT or chest radiography screening and received a screening assessment at baseline, then yearly for 2 more years. Participants were followed for events yearly through questionnaires or National Death Index searches.

We obtained data for the NLST from the National Cancer Institute (NCI)^16^ and used the follow-up time data as recommended in the NCI’s NLST User Guide.^16^ As in the original NLST analyses, we used an event cutoff date of December 31, 2009, for lung cancer–specific mortality and January 15, 2009, for all-cause mortality. After the initial publication of mortality analyses from the NLST,^15^ data completeness was improved, resulting in slightly higher event counts in the updated data available for our analyses (3929 deaths among 53 452 randomized individuals; median 6.5 years of follow-up for lung cancer mortality and 5.5 years of follow-up for all-cause mortality).

#### Target Population

NHIS is a cross-sectional household interview survey conducted every year by the National Center for Health Statistics at the Centers for Disease Control and Prevention.^17,18,19^ NHIS uses a multistage cluster sample design to select households representative of the civilian noninstitutionalized US population.^18^

In certain years, NHIS administers the Cancer Control Supplement (CCS), a supplemental survey on cancer-related health behaviors, screening, and risk assessment, to 1 adult randomly sampled from each selected household.^17^ Information on smoking history for former smokers is available only in years when the CCS is administered (eg, data are available from 2005, 2010, and 2015). We obtained NHIS data from the IPUMS USA database.^14^

We implemented the NLST eligibility criteria in the 2010 NHIS survey to select a target population of US adults to whom the NLST results may be applied, based on meeting the trial’s major eligibility criteria (henceforth referred to as the *target population*). We used the 2010 NHIS survey because it included the CCS supplement and overlapped with the end of follow-up in the NLST,^15^ taking place before CT screening for lung cancer became more widespread following the 2013 USPSTF recommendation.^20^

We provide a detailed mapping of our implementation of the NLST eligibility criteria in NHIS in eTable 1 in Supplement 1. Briefly, we restricted the NHIS sample to ever-smokers (having smoked at least 100 cigarettes in their lifetime) that completed the CCS questions regarding smoking history. Then, we applied the NLST inclusion criteria of age and smoking history to the NHIS sample, along with other exclusion criteria (eg, prior lung cancer diagnosis). Some NLST exclusion criteria, such as requirement for home oxygen supplementation, were not captured in the NHIS data and we did not implement them to select the target population sample. Thus, our analyses transport estimates of the effects of screening on mortality to a target population that meets the major eligibility criteria of the NLST but is slightly broader than the population of trial-eligible individuals.^12^

#### Study Design

Our goal was to transport estimates of the effects of screening from the NLST to the target population represented in the NHIS data.^21^ Because sampling into the NLST and the NHIS was conducted independently, we appended the NLST and NHIS data to form a composite data set that included covariate, assigned screening strategy, and outcome from the NLST and only covariate information from the NHIS.^22^ We used this composite data set to compare the covariate distribution between the NLST and the nationally representative target population and to transport estimates from the NLST to the target population. We mapped covariates from the NLST to NHIS, including demographic characteristics (age, sex, race, ethnicity, and body mass index [calculated as weight in kilograms divided by height in meters squared]), smoking history (current or former smoker and pack-years), comorbidities (asthma, diabetes, emphysema, heart disease, hypertension, and stroke), education, and marital status. These covariates had been included in lung cancer risk models^23,24,25,26^ and were available in both the NLST and NHIS data. Participants in both the NLST and NHIS self-reported their race and ethnicity.

### Statistical Analysis

#### Descriptive Statistics and Comparisons Between Populations

We calculated descriptive statistics for covariates in the NLST and the NHIS sample of the target population and compared the distribution of the covariates between the 2 populations using standardized mean differences or, for discrete variables with multiple categories,^27^ Mahalanobis distances. In these comparisons, we weighted individuals in the NHIS sample using the survey-sampling weights so that the weighted sample represented the target population and gave individuals in NLST weights equal to 1. Absolute standardized mean differences greater than 0.20 suggest a strong imbalance in the covariate distribution between the NLST and the target population^28^; differences of 0.10 suggest a lesser imbalance.^29^

#### Estimating Mortality Rates and Comparing Screening Strategies

We estimated the lung cancer–specific and all-cause mortality rates, along with their differences and ratios, for low-dose CT and chest radiography screening in the NLST (trial only analyses) and in the target population (transportability analyses). In trial only analyses, we used Poisson regression with death as the outcome and assignment as the predictor, with follow-up time as an offset.

#### Transportability Analyses

We used inverse odds of trial participation weighting methods,^21^ accounting for the survey-design of the NHIS.^30^ Similar weighting methods were recently used to transport estimates across NLST centers and to artificial populations^31,32^; instead, we used the methods to learn about a nationally representative target population. Transportability analyses standardize (via weighting) the results of the NLST to the target population by accounting for differences in the distribution of baseline characteristics between the two populations.^13^ We formed “transportability weights” by estimating the probability of trial participation and probability of screening assignment in the trial, conditional on the baseline covariates (Table 1). Next we used Poisson regression, fit only among trial participants, with either lung cancer–specific or all-cause death as the outcome and assignment as the only predictor, weighted by the transportability weights (with weights trimmed to the 99th percentile^33,34^).

**Table 1.** Baseline Characteristics of Participants

Individual characteristics	NLST (n = 51 274)	NHIS (n = 5 739 532)^a^
Demographics		
Age, median (25th percentile and 75th percentile), y	60 (57 and 65)	63 (58 and 67)
Sex		
Female	20 837 (40.6)	2 297 954 (40.0)
Male	30 437 (59.4)	3 441 578 (60.0)
BMI		
<25	14 753 (28.8)	1 751 890 (30.5)
25-30	22 017 (42.9)	2 004 758 (34.9)
30-35	10 188 (19.9)	1 131 103 (19.7)
35-40	3111 (6.1)	411 326 (7.2)
>40	1205 (2.4)	440 455 (7.7)
Race		
African American and Black	2256 (4.4)	408 012 (7.1)
Asian	1061 (2.1)	116 054 (2.0)
Other^b^	1017 (2.0)	68 643 (1.2)
White	46 940 (91.5)	5 146 823 (89.7)
Ethnicity		
Hispanic	763 (1.5)	178 968 (3.1)
Non-Hispanic	50 511 (98.5)	5 560 564 (96.9)
Smoking history		
Former smoker	26 593 (51.9)	2 687 496 (46.8)
Current smoker	24 681 (48.1)	3 052 036 (53.2)
Pack-years		
<40	15 153 (29.6)	1 821 949 (31.7)
41-50	12 514 (24.4)	1 888 045 (32.9)
51-60	7405 (14.4)	785 289 (13.7)
61-70	5174 (10.1)	340 992 (5.9)
71-80	4063 (7.9)	266 294 (4.6)
81-90	2812 (5.5)	230 930 (4.0)
90-100	1556 (3.0)	146 757 (2.6)
>100	2597 (5.1)	259 276 (4.5)
Comorbidities		
Asthma	4893 (9.5)	710 557 (12.4)
Diabetes	4960 (9.7)	1 182 279 (20.6)
Emphysema	3940 (7.7)	559 530 (9.7)
Heart disease	6551 (12.8)	1 025 951 (17.9)
Hypertension	18 223 (35.5)	3 097 819 (54.0)
Stroke	1449 (2.8)	247 997 (4.3)
Education and marital status		
Marital status		
Divorced	10 014 (19.5)	1 136 085 (19.8)
Married or living as married	34 477 (67.2)	3 676 589 (64.1)
Never married	2339 (4.6)	376 426 (6.6)
Separated	641 (1.3)	119 652 (2.1)
Widowed	3803 (7.4)	430 780 (7.5)
Education		
Less than high school	3109 (6.1)	1 105 670 (19.3)
High school graduate	19 732 (38.5)	2 211 463 (38.5)
Some college/Associate’s degree	12 084 (23.6)	1 562 587 (27.2)
Bachelor’s degree or above	16 349 (31.9)	859 812 (15.0)

Abbreviations: NHIS, National Health Interview Survey; NLST, National Lung Screening Trial.

^a^ For NHIS, this number is weighted by the survey weights. It corresponds to 685 unweighted NHIS participants.

^b^ Includes American Indian and Alaskan Native, Native Hawaiian and other Pacific Islander, or more than 1 race.

To interpret the estimates from transportability analyses as estimates of the effectiveness of the screening interventions in the target population we make 2 key assumptions,^12,21^ in addition to those required for the trial only analyses.^35^ First, we assume transportability (exchangeability) of the effects of screening over trial participation status given the measured covariates^21^; informally, this transportability assumption means that the covariates used in the model for the probability of trial participation are sufficient to adjust for selective participation into the trial. Second, we assume that every pattern of these covariates in the target population has a nonzero probability of being observed in the trial (ie, we assume positivity over trial participation status).

To examine how different covariate sets affected results when estimating the probability of trial participation in transportability analyses, we specified a nested sequence of models for the probability of participation to the screening strategies examined in the trial, starting with a model that only included demographic data, and then adding covariates for smoking history, comorbidities, and education and marital status. We refer to the model that adjusted for all these covariates as the fully adjusted model. In each of these analyses, we used the same covariates to model the probability of assignment in the trial (these models for the probability of assignment are always correctly specified, because there is no confounding in the trial).

We examined whether the fully adjusted model balanced the distribution of the covariates by calculating the standardized mean difference for the data from the NLST, weighted by the transportability weights, against the survey-weighted data from NHIS. We used this balance assessment to improve specification of the participation model (without using outcome information at this stage of the analysis).

#### Stability Analyses, Missing Data, and Sensitivity Analysis

To evaluate the stability of the results to different choices about weight trimming, we repeated all analyses without any trimming and with different levels of trimming (99.9th, 99th, and 95th percentile).^33^ Additionally, we repeated the analyses using a Cox proportional-hazards model instead of Poisson regression.

Our main analyses used observations with complete information on the baseline covariates in the first column of Table 1. Missingness was limited (4.1% of NLST observations and 2.8% of NHIS observations meeting trial eligibility criteria). We did not use treatment or outcome data from NHIS, but in the NLST, treatment assignment information was complete and only 2.6% of NLST participants had withdrawn from the study or lost contact from the study by December 31, 2009. As a stability analysis, we used inverse probability of missingness weighting to adjust for missingness in baseline covariates^36^ under a missing at random assumption.

We compared the baseline characteristics of NHIS samples meeting the NLST eligibility criteria from recent years when the CCS was administered (2005, 2010, and 2015). We used more relaxed criteria for all years when comparing because the 2005 data does not have information on chest CT screening or cancer treatment. The CCS was also administered in 2020, but because the NHIS design was changed in 2019 it is not recommended to compare pre-2019 and post-2019 NHIS samples. We repeated the main transportability analysis (with the fully adjusted model) using NHIS years before screening was recommended by the USPSTF, while the trial was ongoing (2005 and 2010). We repeated this analysis in 2010 with the more relaxed criteria and in an analysis that combined the 2005 and 2010 data.

We conducted a sensitivity analysis to quantify how violations of the transportability assumption would affect our results by estimating the comparative effectiveness of screening strategies for the magnitudes of the association between participation in the NLST and the unobserved potential (counterfactual) outcomes under each screening strategy, given the observed covariates.^37^

Additional details of the statistical analyses, including the sensitivity analysis, are available in eMethods and eFigure in Supplement 1. Data were analyzed from March 2020 to September 2023 using R statistical software version 4.1.3 (R Project for Statistical Computing).^38^

## Results

**Figure 1.**
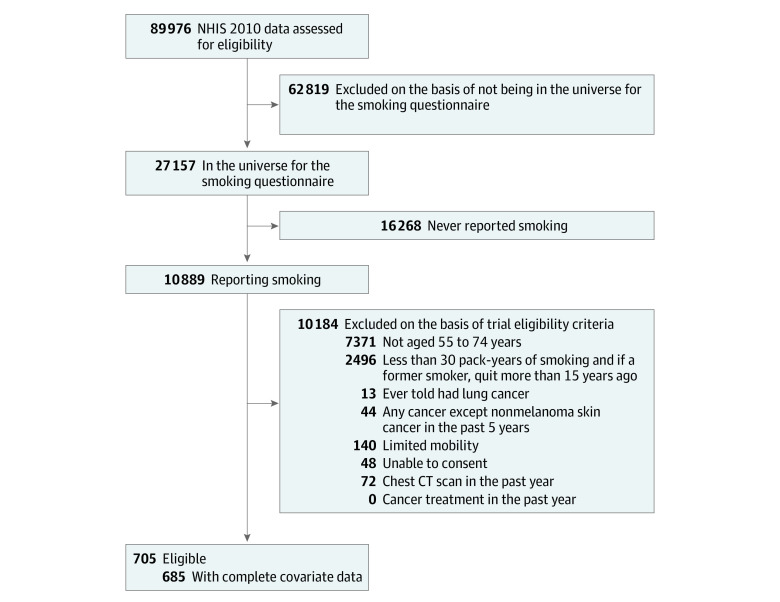
Flow Diagram for Identifying the Target Population Sample in National Health Interview Survey Exclusion criteria are applied in a stepwise order.

The transportability analysis included 51 274 NLST participants and 685 NHIS participants (5 700 000 when survey weighted) that represent the target population. Compared with the target population, NLST participants were younger (median [25th percentile and 75th percentile] age, 60 [57 and 65] years vs 63 [58 and 67] years), had fewer comorbidities (eg, 6551 of 51 274 [12.8%] vs 1 025 951 of 5 739 532 [17.9%] had heart disease), and were more educated (16 349 of 51 274 [31.9%] vs 859 812 of 5 739 532 [15.0%] had a bachelor’s degree or higher). Figure 1 shows the process of identifying NHIS participants for our analyses. Most NHIS participants were excluded because they were not sampled by design into the CCS, which only samples 1 adult from each selected household, for collection of smoking-related information (70% of all unweighted observations in NHIS).

**Figure 2.**
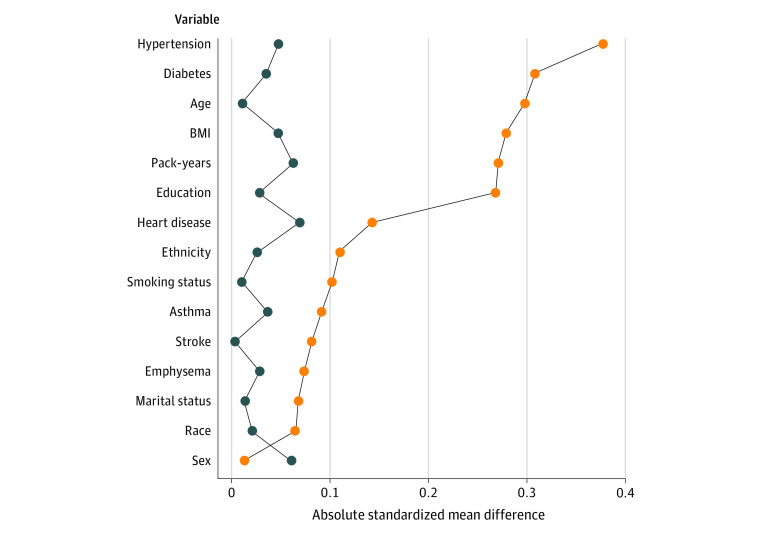
Absolute Standardized Mean Differences Comparing the Trial Sample and the Target Population Before and After Weighting Orange dots indicate unadjusted mean differences before weighting; blue dots, mean differences after weighting.

Age, body mass index, diabetes, hypertension, education and pack-years were strongly imbalanced (absolute standardized mean differences greater than 0.2) between NLST participants and the target population (Table 1). Figure 2 shows absolute standardized mean differences before and after weighting NLST participants by the transportability weights vs the target population, using the fully adjusted model to evaluate covariate balance. After weighting, the absolute standardized mean differences for all covariates indicated that balance was adequate (ie, all absolute standardized mean differences were less than 0.10).

Using the fully adjusted model, the estimated relative lung cancer–specific mortality rate reduction was 18% (95% CI, 1% to 33%) in the target population vs 21% (95% CI, 9% to 32%) in the NLST (Table 2). Using the fully adjusted model, the estimated absolute lung cancer–specific mortality rate reduction was 71 deaths per 100 000 person-years (95% CI, 4 to 138 deaths per 100 000 person-years) in the target population vs 67 deaths per 100 000 person-years (95% CI, 27 to 106 deaths per 100 000 person-years) in the NLST. Using the fully adjusted model, the estimated relative all-cause mortality rate reduction was 6% (95% CI, −2% to 12%) in the target population vs 7% (95% CI, 0% to 12%) in the NLST (eTable 2 in Supplement 1).

**Table 2.** Lung Cancer–Specific Mortality Results for Trial Only and Transportability Analyses for Low-Dose CT vs Chest Radiography

Analysis	Deaths per 100 000 person-years (95% CI)			Rate ratio (95% CI)
	Low-dose CT	Chest radiography	Rate difference	
Trial only (unadjusted)	245 (220 to 272)	312 (283 to 342)	−67 (−106 to −27)	0.79 (0.68 to 0.91)
Nationally representative target population (adjusted)^a^				
Demographics only	287 (256 to 322)	352 (318 to 390)	−65 (−114 to −17)	0.81 (0.70 to 0.95)
Demographics plus smoking history	286 (254 to 323)	352 (316 to 392)	−66 (−117 to −15)	0.81 (0.69 to 0.96)
Demographics, smoking history, and comorbidities	299 (261 to 343)	374 (331 to 423)	−75 (−137 to −14)	0.80 (0.66 to 0.96)
Demographics, smoking history, comorbidities, and education and marital status	315 (272 to 364)	386 (340 to 438)	−71 (−138 to −4)	0.82 (0.67 to 0.99)

Abbreviation: CT, computed tomography.

^a^ Weights in the adjusted analysis are trimmed to the 99th percentile among trial participants. The simplest analysis only adjusts for demographics; the fully adjusted analysis adjusts for demographics, smoking history, comorbidities, and education and marital status.

### Stability Analyses, Missing Data, and Sensitivity Analysis

Lung cancer–specific and all-cause mortality estimates were stable in analyses without trimming or using heavier trimming than in our main analyses (eTables 3 and 4 in Supplement 1), suggesting that our results are not driven by extreme weights. For both outcomes, estimates from Cox proportional-hazards regression agreed with those from Poisson regression (eTables 5 and 6 in Supplement 1). Analyses adjusted for missing baseline covariates produced estimates that were nearly identical to complete case analyses (eTables 7 and 8 in Supplement 1). We found that the demographics characteristics and stability analyses that used different years of NHIS were also similar to our main results (eTables 9-11 in Supplement 1).

In sensitivity analyses, the rate ratio for lung cancer–specific mortality was similar to the results reported in the main analysis, even under strong violations of the transportability assumption, provided that the violations were of similar magnitude for lung cancer–specific mortality under both screening strategies (eFigure 1 in Supplement 1). In contrast, under violations of the assumption reflecting differential selection into the trial, our results could either underestimate or overestimate the benefit of screening compared with the main analysis. For instance, the benefit of low-dose CT screening would be underestimated in our analyses if the trial preferentially enrolled people who had worse lung cancer–specific mortality with low-dose CT vs chest radiography screening compared with the national population. To change our qualitative conclusions about the comparative effectiveness of these screening strategies, one would have to assume moderately strong and differential selection into the trial (compared with the target population) of individuals who would benefit more (or be harmed less) by low-dose CT screening compared with chest radiography screening.

## Discussion

In our analyses, the estimated effects of low-dose CT screening compared with chest radiography in a nationally representative target population were similar to those in the NLST, particularly on the relative scale. However, after adjusting for differences in measured characteristics between the NLST sample and the target population, our estimates were less precise than those obtained in the NLST. The increased uncertainty was due to large differences in age, comorbidities, and education between the 2 populations. Such differences in baseline characteristics have been previously reported,^9,10,11^ but our work is the first to account for them when estimating the impact of screening for a clinically relevant target population.

Our findings provide support for the USPSTF recommendation in favor of low-dose CT screening for adults ages 55 to 80 years who have a 30 pack-year smoking history and currently smoke or have quit within the past 15 years, with the caveat that extrapolation to a nationally representative target population is subject to additional uncertainty. The recent USPSTF recommendation also included adults with a smoking history of 20 to 30 pack-years, in addition to those with a history of 30 pack-years or more, and lowered the age range to include adults ages 50 to 80 years. Our approach cannot use data from the NLST to estimate the impact among individuals with a smoking history of fewer than 30 pack-years or individuals younger than 55 years old (without additional extrapolation assumptions) because such individuals were not eligible for the trial. Estimates for a broader target population may be obtained in future work combining information from multiple trials,^39^ including those with eligibility criteria that allowed for the lower number of pack-years or younger age at enrollment (eg, the Nederlands-Leuvens Screening Onderzoek [NELSON] trial^40^).

### Limitations

Our findings need to be interpreted in view of the limitations of our approach. First, our analyses can only account for variables that were included in the model for the probability of trial participation^21^ and require that model to be correctly specified. We found that our results were stable across different specifications, such as when we added additional variables considered important in lung cancer risk models. This provides some indirect evidence that our conclusions are less likely to be driven by unmeasured variables or model misspecification. Furthermore, we found that our results about the rate ratios for lung cancer-specific mortality were stable in sensitivity analyses that examined violations of the transportability assumption, except when the assumption was differentially violated between the different screening strategies. Second, our analyses estimate the effectiveness of screening policies under adherence similar to that observed in the NLST.^41^ Yet, adherence to screening in the NLST was approximately 90%,^15^ whereas adherence to screening in community settings is lower.^42^ For example, recent work reported that adherence to repeated rounds of lung cancer screening was low among the first 1 000 000 patients enrolled in a national lung cancer screening registry.^43^ Thus, our results may reflect best-case estimates and highlight the importance of interventions to improve adherence to effective screening programs. Our estimates can be used to inform simulation studies to examine screening effectiveness under different levels of adherence to screening. Third, we cannot adjust for the evolution in screening strategies and subsequent diagnostic or therapeutic interventions since the completion of the NLST.^12^ Because the impact of screening on clinical outcomes is mediated by screening-directed interventions, future decision modeling investigations may combine our transportability approach with evidence about the effectiveness of novel screening-directed interventions, including applying our approach to intermediate outcomes that mediate the effects of screening on mortality (eg, stage shift, complications, and overdiagnosis rates). In addition, CT screening performance can be improved with the introduction of new technologies, such as computer-aided detection.^44^ Such improvements mean that our results underestimate the comparative effectiveness of newer CT-based screening approaches in the target population. Fourth, as in the original analysis of the NLST, we did not adjust for competing events when estimating lung cancer–specific mortality. Nevertheless, our lung cancer–specific analyses adjusted for several comorbidities that may explain differences in competing risks; furthermore, our all-cause mortality analyses are not subject to competing events.

## Conclusion

This comparative effectiveness study suggests that the effects of low-dose CT screening compared with chest radiography in the target population were similar to those in the NLST, particularly on the relative scale. Estimates of effectiveness in the target population were more uncertain than those in the NLST due to differences in baseline characteristics between the target population and the trial.

## Notes

Supplement 1.
eMethods.
**eTable 1.** List of Eligibility Criteria From the NLST Protocol and Corresponding Implementation in NHIS **eTable 2.** All-Cause Mortality Results for the Trial Only (Unadjusted) and Transportability Analyses (Adjusting for Different Covariate Sets) for Low-Dose CT vs Chest Radiography, With Weights in the Adjusted Analysis Trimmed to the 99th Percentile Among Trial Participants **eTable 3.** Lung Cancer–Specific Mortality Results for the Trial Only (Unadjusted) and Transportability Analyses (Adjusting for Different Covariate Sets) for Low-Dose CT vs Chest Radiography, With Untrimmed Weights or Weights Trimmed to the 99.9th, 99th, and 95th Percentile Among Trial Participants **eTable 4.** All-Cause Mortality Results for the Trial Only (Unadjusted) and Transportability Analyses (Adjusting for Different Covariate Sets) for Low-Dose CT vs Chest Radiography, With Untrimmed Weights or Weights Trimmed to the 99.9th, 99th, and 95th Percentile Among Trial Participants **eTable 5.** Lung Cancer–Specific Mortality Results for the Trial Only (Unadjusted) and Transportability Analyses (Adjusting for Different Covariate Sets) for Low-Dose CT vs Chest Radiography, Using a Cox Proportional-Hazards Model **eTable 6.** All-Cause Mortality Results for the Trial Only (Unadjusted) and Transportability Analyses (Adjusting for Different Covariate Sets) for Low-Dose CT vs Chest Radiography, Using a Cox Proportional-Hazards Model **eTable 7.** Lung Cancer–Specific Results for the Trial Only (Unadjusted) and Transportability Analyses (Adjusting for Different Covariate Sets) for Low-Dose CT vs Chest Radiography When Adjusting for Missingness, With Weights in the Transportability Analysis Trimmed to 99% **eTable 8.** All-Cause Mortality Results for the Trial Only (Unadjusted) and Transportability Analyses (Adjusting for Different Covariate Sets) for Low-Dose CT vs Chest Radiography When Adjusting for Missingness, With Weights in the Transportability Analysis Trimmed to 99% **eTable 9.** Comparison of NHIS Baselines Across Years Using More Relaxed Eligibility Criteria, Weighted by Survey Weights **eTable 10.** Lung Cancer–Specific Mortality Results for the Transportability Analyses Using the Fully Adjusted Model, With Weights in the Transportability Analysis Trimmed to 99% **eTable 11.** All-Cause Mortality Results for Using the Fully Adjusted Model, With Weights in the Transportability Analysis Trimmed to 99% **eFigure.** Sensitivity Analysis Results, on the Rate Ratio Scale for Lung Cancer Mortality, for Violations of the Transportability Assumption
eReferences.

Supplement 2.
Data Sharing Statement
