# Supplementary material for: Comparing Lung Cancer Screening Strategies in a Nationally Representative US Population Using Transportability Methods for the National Lung Cancer Screening Trial
Source: JAMA Netw Open. 2024 Jan 30;7(1):e2346295. doi: 10.1001/jamanetworkopen.2023.46295 (PMC10828917; doi:10.1001/jamanetworkopen.2023.46295)
Supplement: Supplement 1. — eMethods. eTable 1. List of Eligibility Criteria From the NLST Protocol and Corresponding Implementation in NHIS eTable 2. All-Cause Mortality Results for the Trial Only (Unadjusted) and Transportability Analyses (Adjusting for Different Covariate Sets) for Low-Dose CT vs Chest Radiography, With Weights in the Adjusted Analysis Trimmed to the 99th Percentile Among Trial Participants eTable 3. Lung Cancer–Specific Mortality Results for the Trial Only (Unadjusted) and Transportability Analyses (Adjusting for Different Covariate Sets) for Low-Dose CT vs Chest Radiography, With Untrimmed Weights or Weights Trimmed to the 99.9th, 99th, and 95th Percentile Among Trial Participants eTable 4. All-Cause Mortality Results for the Trial Only (Unadjusted) and Transportability Analyses (Adjusting for Different Covariate Sets) for Low-Dose CT vs Chest Radiography, With Untrimmed Weights or Weights Trimmed to the 99.9th, 99th, and 95th Percentile Among Trial Participants eTable 5. Lung Cancer–Specific Mortality Results for the Trial Only (Unadjusted) and Transportability Analyses (Adjusting for Different Covariate Sets) for Low-Dose CT vs Chest Radiography, Using a Cox Proportional-Hazards Model eTable 6. All-Cause Mortality Results for the Trial Only (Unadjusted) and Transportability Analyses (Adjusting for Different Covariate Sets) for Low-Dose CT vs Chest Radiography, Using a Cox Proportional-Hazards Model eTable 7. Lung Cancer–Specific Results for the Trial Only (Unadjusted) and Transportability Analyses (Adjusting for Different Covariate Sets) for Low-Dose CT vs Chest Radiography When Adjusting for Missingness, With Weights in the Transportability Analysis Trimmed to 99% eTable 8. All-Cause Mortality Results for the Trial Only (Unadjusted) and Transportability Analyses (Adjusting for Different Covariate Sets) for Low-Dose CT vs Chest Radiography When Adjusting for Missingness, With Weights in the Transportability Analysis Trimmed to 99% eTable 9. Comparison of NHIS Baselines [file jamanetwopen-e2346295-s001.pdf]

Supplemental Online Content

Robertson SE, Joyce NR, Steingrimsson JA, et al. Estimating the effects of lung cancer screening strategies in a nationally representative US population. *JAMA Netw Open.* 2023;6(12):e2346295. doi:10.1001/jamanetworkopen.2023.46295

eMethods.

eTable 1. List of Eligibility Criteria from the NLST Protocol and Corresponding Implementation in NHIS

eTable 2. All-Cause Mortality Results for the Trial Only (Unadjusted) and Transportability Analyses (Adjusting for Different Covariate Sets) for Low-Dose CT vs Chest Radiography, With Weights in the Adjusted Analysis Trimmed to the 99th Percentile Among Trial Participants

eTable 3. Lung Cancer–Specific Mortality Results for the Trial Only (Unadjusted) and Transportability Analyses (Adjusting for Different Covariate Sets) for Low-Dose CT vs Chest Radiography, With Untrimmed Weights or Weights Trimmed to the 99.9th, 99th, and 95th Percentile Among Trial Participants

eTable 4. All-Cause Mortality Results for the Trial Only (Unadjusted) and Transportability Analyses (Adjusting for Different Covariate Sets) for Low-Dose CT vs Chest Radiography, With Untrimmed Weights or Weights Trimmed to the 99.9th, 99th, and 95th Percentile Among Trial Participants

eTable 5. Lung Cancer–Specific Mortality Results for the Trial Only (Unadjusted) and Transportability Analyses (Adjusting for Different Covariate Sets) for Low-Dose CT vs Chest Radiography, Using a Cox Proportional-Hazards Model

eTable 6. All-Cause Mortality Results for the Trial Only (Unadjusted) and Transportability Analyses (Adjusting for Different Covariate Sets) for Low-Dose CT vs Chest Radiography, Using a Cox Proportional-Hazards Model

eTable 7. Lung Cancer–Specific Results for the Trial Only (Unadjusted) and Transportability Analyses (Adjusting for Different Covariate Sets) for Low-Dose CT vs Chest Radiography When Adjusting for Missingness, With Weights in the Transportability Analysis Trimmed to 99%

eTable 8. All-Cause Mortality Results for the Trial Only (Unadjusted) and Transportability Analyses (Adjusting for Different Covariate Sets) for Low-Dose CT vs

© 2023 Robertson SE et al. *JAMA Network Open.*

Chest Radiography When Adjusting for Missingness, With Weights in the Transportability Analysis Trimmed to 99%

eTable 9. Comparison of NHIS Baselines Across Years Using More Relaxed Eligibility Criteria, Weighted by Survey Weights

eTable 10. Lung Cancer–Specific Mortality Results for the Transportability Analyses Using the Fully Adjusted Model, With Weights in the Transportability Analysis Trimmed to 99%

eTable 11. All-Cause Mortality Results for Using the Fully Adjusted Model, With Weights in the Transportability Analysis Trimmed to 99%

eFigure. Sensitivity Analysis Results, on the Rate Ratio Scale for Lung Cancer Mortality, for Violations of the Transportability Assumption

eReferences.

This supplemental material has been provided by the authors to give readers additional information about their work.

© 2023 Robertson SE et al. *JAMA Network Open.*

eMethods.

Transportability weights: We obtained weights for the transportability analyses by estimating the probability of trial participation and the probability of assignment to each screening strategy in the trial, conditional on the covariates in Table 1 of the main text. To avoid extreme weights and improve balance, we chose to collapse the following multi-category variables from Table 1 into binary variables: race was coded as white versus non-white, marital status was coded as married/living as married versus all others, and education was coded as any college versus no college. To form the transportability weights, we calculated the inverse of the estimated odds of trial participation.[1] To estimate the probability of trial participation and the probability of assignment to each screening strategy in the trial, we used logistic regression models. Specifically, the probability of trial participation was estimated using a weighted logistic regression with weights for the NHIS participants set to the survey sample weights and weights for the NLST participants set to 1. The probability of assignment to each screening strategy in the trial was estimated using unweighted logistic regression using the trial data alone. To estimate measures of incidence and measures of association in the target population, we used outcome regressions with treatment as the only predictor in the model and obtained standard errors for the model parameters using the robust estimator of the sampling variance.[1]

Examining the transportability weights: To examine the transportability weights, we calculated the ratio of the sum of the weights over all individuals in the composite dataset, divided by the number of individuals in the target population (after using the survey weights).[1] Values of this statistic that are meaningfully different from 1 suggest violations of the assumption that every pattern of these covariates in the target population has a non-zero probability of being observed in the trial (i.e., positivity over trial participation status) or misspecification of the model for the probability of participation. In our main analysis (using the fully adjusted model with weights trimmed to the 99th percentile of their empirical distribution), the value of the diagnostic was 1.05, suggesting a lack of violations.

Handling missing data: The analyses presented in the main text used observations with complete information on the baseline covariates listed in Table 1 of the main text. Missingness was limited for covariates; no assignment data was missing in the trial. As a stability analysis, we used inverse probability of missingness weighting to adjust for missingness in any baseline covariates.[2] In the model for the probability of missingness among NLST participants, we included the screening intervention, outcome, and all of the baseline covariates used in the fully adjusted model with complete observations (age, sex, pack-years, current smoking status) as predictors. In the model for the probability of missingness among NHIS participants, we included baseline covariates used in the fully adjusted model with complete observations (age, sex, ethnicity, pack-years, current smoking status, body mass index, race, and diabetes) as predictors. We repeated the trial-only analysis and transportability analyses incorporating the missingness weights.

Sensitivity analyses: We conducted sensitivity analyses to examine how violations of the transportability assumption would affect our results. These sensitivity analyses estimate the comparative effectiveness of screening strategies under different magnitudes of assumption

© 2023 Robertson SE et al. *JAMA Network Open.*

violations by varying the strength of association between participation in the NLST and the potential outcomes under each screening strategy, conditional on the observed covariates. We used the NLST and 2010 NHIS data to examine the sensitivity of the transportability analysis for lung cancer-specific mortality on the rate ratio scale. We modified a global sensitivity analysis approach[3,4] that uses an odds of selection model with sensitivity parameters that express the conditional association between trial participation and the (unobservable) potential outcomes under the two screening strategies compared in the trial, given the measured covariates. [5,6] We tailored this approach to use survey-weighted data by accounting for covariate-dependent sampling probabilities.[2-4] We estimated the effectiveness of screening in the target population using the approach described in the main text – Poisson regression, weighted by the transportability weights – with the transportability weights were re-estimated for different values of the sensitivity parameters. As in the main analysis, we used the fully adjusted model when forming the transportability weights and trimmed the weights to the 99th percentile.

Different values of the sensitivity parameters express different beliefs about the association between trial participation and the potential outcome under each treatment (separately for each screening group in the trial) on the logit scale, given the measured covariates included in the analysis. We examined values of the sensitivity parameters ranging from -0.30 (denoting that lung cancer-specific mortality under the screening or control intervention made participation more likely) to 0.30 (denoting that lung cancer-specific mortality under the screening or control intervention made participation less likely).

The results of the sensitivity analysis are shown in the contour plot of **eFigure 1**. In this plot, the black "x" corresponds to the point estimate of the main analysis, when the sensitivity parameters are set to 0 (i.e., when the transportability assumption holds). The estimated effectiveness of screening varies according to the value of the sensitivity parameters. For sensitivity parameter values that correspond to the diagonal that runs from the bottom left to the top right (i.e., the diagonal where the "x" falls; teal blue color), the results are close to those from our main analysis for lung cancer-specific mortality, even for fairly strong violations of the assumption. That is the case because for this set of potential violations of the assumption of selection into the trial is not differentially associated with the potential outcomes for lung cancer-specific mortality under each screening strategy (e.g., they reflect situations in which the risk of the outcome in the target population may be lower or higher than the population enrolled in the trial, but not differentially between different screening strategies).

For sensitivity parameter values corresponding to the region below the diagonal (blue color), the main analysis results overestimate the benefit of low-dose CT screening. This would happen, for example, if the trial preferentially enrolled people who have reduced lung cancer-specific mortality with low-dose CT screening versus chest radiography screening (because of unmeasured variables) compared to the national population. For sensitivity parameter values corresponding to the region above the diagonal (yellow color), our main analysis results would underestimate the benefit of low-dose CT screening. This would happen, for example, if the trial preferentially enrolled people who had increased lung cancer-specific mortality with low-dose CT screening versus chest radiography screening (because of unmeasured variables) compared to the national population.

In summary, under violations of the transportability assumption reflecting differential selection into the trial, our main analysis results could either underestimate or overestimate the benefit from low-dose CT screening compared with chest radiography screening. To change our qualitative conclusions about the comparative effectiveness of these screening strategies, one

© 2023 Robertson SE et al. *JAMA Network Open.*

would have to assume moderately strong and differential selection into the trial (compared to the target population) of individuals who would benefit more (or be harmed less) by low-dose CT screening compared with chest radiography screening.

© 2023 Robertson SE et al. *JAMA Network Open*.

eTable 1. List of Eligibility Criteria from the NLST Protocol and Corresponding Implementation in NHIS

**NLST eligibility**	**Implementation in NHIS [data source]**
Inclusion criteria	
Age 55-74 years	Same [core NHIS]
30 or more pack-years of cigarette smoking history	Same [core NHIS and CCS]
Former smokers: quit smoking within the previous 15 years	Same [CCS]
Signed informed consent form	No trouble remembering
Exclusion criteria	
Inability to lie on the back with arms raised above the head.	Excluded individuals unable to reach overhead or bed days greater than 30 or reported needed help with the activities of daily living [core NHIS]
Metallic implants or metallic devices in the chest or back, such as pacemakers or Harrington fixation rods	Not available
History of lung cancer	Same [core NHIS]
Treatment for, or evidence of, any cancer other than nonmelanoma skin cancer or carcinoma in situ (with the exception of transitional cell carcinoma in situ or bladder carcinoma in situ) in the 5 years prior to eligibility assessment	Excluded individuals who reported receiving treatment for cancer in the past year [core NHIS]; excluded individuals reporting cancer other than nonmelanoma skin cancer in the 5 years prior [CCS]
History of removal of any portion of the lung, excluding needle biopsy	Not available
Requirement for home oxygen supplementation	Not available
Participation in another cancer screening trial	Not available
Participation in a cancer prevention trial other than smoking cessation programs.	Not available
Unexplained weight loss of more than 15 lbs in 12 months prior to eligibility assessment, or unexplained hemoptysis	Not available
Pneumonia or acute respiratory infection treated with antibiotics in the 12 weeks prior to eligibility assessment	Not available
Chest CT examination in the 18 months prior to eligibility assessment	Excluded individuals with any CT scan in the past year [CCS]

© 2023 Robertson SE et al. *JAMA Network Open.*

eTable 2. All-Cause Mortality Results for the Trial Only (Unadjusted) and Transportability Analyses (Adjusting for Different Covariate Sets) for Low-Dose CT vs Chest Radiography, With Weights in the Adjusted Analysis Trimmed to the 99th Percentile Among Trial Participants

Analysis	Low-dose CT	Chest radiography	Rate ratio	Rate difference
Trial-only (unadjusted)	1126 (1076, 1179)	1205 (1153, 1260)	0.93 (0.88, 1.00)	-79 (-153, -4)
Nationally representative target population, adjusting for:				
Demographics	1342 (1277, 1411)	1431 (1363, 1501)	0.94 (0.88, 1.01)	-89 (-185, 7)
+ Smoking history	1337 (1269, 1409)	1425 (1354, 1499)	0.94 (0.87, 1.01)	-88 (-188, 13)
+ Comorbidities	1541 (1453, 1633)	1631 (1541, 1725)	0.94 (0.87, 1.02)	-90 (-219, 39)
+ Education/marital status	1577 (1484, 1675)	1673 (1579, 1773)	0.94 (0.87, 1.02)	-96 (-232, 40)

© 2023 Robertson SE et al. *JAMA Network Open.*

eTable 3. Lung Cancer-Specific Mortality Results for the Trial Only (Unadjusted) and Transportability Analyses (Adjusting for Different Covariate Sets) for Low-Dose CT vs Chest Radiography, With Untrimmed Weights or Weights Trimmed to the 99.9th, 99th, and 95th Percentile Among Trial Participants

Analysis	Trim level	Low-dose CT	Chest radiography	Rate ratio	Rate difference
Trial-only (unadjusted)	Not applicable	245 (220, 272)	312 (283, 342)	0.79 (0.68, 0.91)	-67 (-106, -27)
Nationally representative target population, adjusting for:					
Demographics	Untrimmed	289 (257, 326)	353 (318, 391)	0.82 (0.70, 0.96)	-63 (-114, -13)
Demographics	0.999	289 (256, 325)	352 (318, 391)	0.82 (0.70, 0.96)	-64 (-114, -14)
Demographics	0.99	287 (256, 322)	352 (318, 390)	0.81 (0.70, 0.95)	-65 (-114, -17)
Demographics	0.95	287 (257, 322)	354 (321, 392)	0.81 (0.70, 0.94)	-67 (-115, -19)
+ Smoking history	Untrimmed	288 (254, 327)	350 (314, 390)	0.82 (0.70, 0.97)	-62 (-114, -9)
+ Smoking history	0.999	288 (254, 326)	351 (315, 391)	0.82 (0.70, 0.97)	-62 (-115, -10)
+ Smoking history	0.99	286 (254, 323)	352 (316, 392)	0.81 (0.69, 0.96)	-66 (-117, -15)
+ Smoking history	0.95	286 (254, 322)	354 (319, 393)	0.81 (0.69, 0.95)	-68 (-118, -18)
+ Comorbidities	Untrimmed	312 (262, 371)	375 (329, 426)	0.83 (0.67, 1.03)	-63 (-135, 10)
+ Comorbidities	0.999	303 (261, 352)	376 (330, 427)	0.81 (0.66, 0.98)	-73 (-139, -6)
+ Comorbidities	0.99	299 (261, 343)	374 (331, 423)	0.80 (0.66, 0.96)	-75 (-137, -14)
+ Comorbidities	0.95	293 (257, 333)	367 (328, 411)	0.80 (0.67, 0.95)	-74 (-130, -18)
+ Education/marital status	Untrimmed	336 (271, 415)	389 (339, 447)	0.86 (0.67, 1.11)	-53 (-143, 36)
+ Education/marital status	0.999	319 (273, 372)	391 (340, 449)	0.82 (0.66, 1.01)	-72 (-145, 2)
+ Education/marital status	0.99	315 (272, 364)	386 (340, 438)	0.82 (0.67, 0.99)	-71 (-138, -4)
+ Education/marital status	0.95	301 (264, 343)	375 (334, 421)	0.80 (0.67, 0.96)	-74 (-133, -16)

© 2023 Robertson SE et al. *JAMA Network Open.*

eTable 4. All-Cause Mortality Results for the Trial Only (Unadjusted) and Transportability Analyses (Adjusting for Different Covariate Sets) for Low-Dose CT vs Chest Radiography, With Untrimmed Weights or Weights Trimmed to the 99.9th, 99th, and 95th Percentile Among Trial Participants

Analysis	Trim level	Low-dose CT	Chest radiography	Rate ratio	Rate difference
Trial-only (unadjusted)	Not applicable	1126 (1076, 1179)	1205 (1153, 1260)	0.93 (0.88, 1.00)	-79 (-153, -4)
Nationally representative target population, adjusting for:					
Demographics	Untrimmed	1363 (1294, 1435)	1447 (1377, 1522)	0.94 (0.88, 1.01)	-85 (-186, 16)
Demographics	0.999	1361 (1293, 1434)	1445 (1375, 1518)	0.94 (0.88, 1.01)	-83 (-184, 17)
Demographics	0.99	1342 (1277, 1411)	1431 (1363, 1501)	0.94 (0.88, 1.01)	-89 (-185, 7)
Demographics	0.95	1319 (1257, 1384)	1415 (1350, 1483)	0.93 (0.87, 1.00)	-96 (-188, -4)
+ Smoking history	Untrimmed	1360 (1286, 1438)	1434 (1361, 1511)	0.95 (0.88, 1.02)	-74 (-180, 32)
+ Smoking history	0.999	1356 (1284, 1432)	1436 (1363, 1512)	0.94 (0.88, 1.02)	-80 (-185, 25)
+ Smoking history	0.99	1337 (1269, 1409)	1425 (1354, 1499)	0.94 (0.87, 1.01)	-88 (-188, 13)
+ Smoking history	0.95	1309 (1245, 1377)	1401 (1334, 1471)	0.93 (0.87, 1.00)	-92 (-187, 3)
+ Comorbidities	Untrimmed	1617 (1508, 1734)	1671 (1571, 1778)	0.97 (0.88, 1.06)	-54 (-208, 99)
+ Comorbidities	0.999	1586 (1488, 1691)	1667 (1569, 1772)	0.95 (0.87, 1.04)	-81 (-224, 62)
+ Comorbidities	0.99	1541 (1453, 1633)	1631 (1541, 1725)	0.94 (0.87, 1.02)	-90 (-219, 39)
+ Comorbidities	0.95	1471 (1394, 1553)	1568 (1488, 1652)	0.94 (0.87, 1.01)	-96 (-210, 18)
+ Education/marital status	Untrimmed	1660 (1539, 1790)	1723 (1612, 1841)	0.96 (0.87, 1.07)	-63 (-233, 107)
+ Education/marital status	0.999	1619 (1516, 1730)	1712 (1607, 1825)	0.95 (0.86, 1.04)	-93 (-246, 59)
+ Education/marital status	0.99	1577 (1484, 1675)	1673 (1579, 1773)	0.94 (0.87, 1.02)	-96 (-232, 40)
+ Education/marital status	0.95	1493 (1413, 1577)	1596 (1514, 1683)	0.94 (0.87, 1.01)	-104 (-221, 14)

© 2023 Robertson SE et al. *JAMA Network Open.*

eTable 5. Lung Cancer-Specific Mortality Results for the Trial Only (Unadjusted) and Transportability Analyses (Adjusting for Different Covariate Sets) for Low-Dose CT vs Chest Radiography, Using a Cox Proportional-Hazards Model

**Analysis**	**Rate ratio**
Trial-only (unadjusted)	0.79 (0.68, 0.91)
Nationally representative target population, adjusting for:	
Demographics	0.81 (0.70, 0.95)
+ Smoking history	0.81 (0.69, 0.96)
+ Comorbidities	0.94 (0.87, 1.02)
+ Education/marital status	0.82 (0.87, 0.99)

© 2023 Robertson SE et al. *JAMA Network Open.*

eTable 6. All-Cause Mortality Results for the Trial Only (Unadjusted) and Transportability Analyses (Adjusting for Different Covariate Sets) for Low-Dose CT vs Chest Radiography, Using a Cox Proportional-Hazards Model

**Analysis**	**Rate ratio**
Trial-only (unadjusted)	0.93 (0.88, 1.00)
Nationally representative target population, adjusting for:	
Demographics	0.94 (0.88, 1.01)
+ Smoking history	0.94 (0.87, 1.01)
+ Comorbidities	0.94 (0.87, 1.02)
+ Education/marital status	0.94 (0.87, 1.02)

© 2023 Robertson SE et al. *JAMA Network Open.*

eTable 7. Lung Cancer-Specific Results for the Trial Only (Unadjusted) and Transportability Analyses (Adjusting for Different Covariate Sets) for Low-Dose CT vs Chest Radiography When Adjusting for Missingness, With Weights in the Transportability Analysis Trimmed to 99%

Analysis	Low-dose CT	Chest radiography	Rate ratio	Rate difference
Trial-only (unadjusted)	246 (221, 274)	312 (284, 343)	0.79 (0.68, 0.91)	-66 (-106, -27)
Nationally representative target population, adjusting for:				
Demographics	287 (256, 322)	353 (318, 390)	0.81 (0.70, 0.95)	-66 (-114, -17)
+ Smoking history	285 (252, 321)	352 (316, 392)	0.81 (0.69, 0.95)	-67 (-118, -16)
+ Comorbidities	297 (259, 341)	375 (331, 423)	0.79 (0.66, 0.95)	-77 (-139, -16)
+ Education/marital status	313 (271, 362)	386 (340, 438)	0.81 (0.67, 0.98)	-73 (-139, -6)

© 2023 Robertson SE et al. *JAMA Network Open.*

eTable 8. All-Cause Mortality Results for the Trial Only (Unadjusted) and Transportability Analyses (Adjusting for Different Covariate Sets) for Low-Dose CT vs Chest Radiography When Adjusting for Missingness, With Weights in the Transportability Analysis Trimmed to 99%

Analysis	Low-dose CT	Chest radiography	Rate ratio	Rate difference
Trial-only (unadjusted)	1135 (1084, 1188)	1215 (1162, 1270)	0.93 (0.88, 1.00)	-80 (-155, -5)
Nationally representative target population, adjusting for:				
Demographics	1351 (1285, 1420)	1442 (1374, 1513)	0.94 (0.87, 1.00)	-91 (-188, 6)
+ Smoking history	1343 (1275, 1415)	1432 (1361, 1507)	0.94 (0.87, 1.01)	-89 (-190, 12)
+ Comorbidities	1546 (1458, 1639)	1638 (1549, 1733)	0.94 (0.87, 1.02)	-93 (-222, 36)
+ Education/marital status	1582 (1489, 1680)	1680 (1585, 1781)	0.94 (0.87, 1.02)	-98 (-235, 38)

© 2023 Robertson SE et al. *JAMA Network Open.*

eTable 9. Comparison of NHIS Baselines Across Years Using More Relaxed Eligibility Criteria, Weighted by Survey Weights

Individual characteristics	Survey year		
	2005 (n=6,335,267)	2010 (n=6,341,677)	2015 (n=5,432,203)
Demographics			
Age in years	62 [58, 66]	63 [58, 67]	62 [59, 67]
Sex			
Female	2,373,102 (37.5)	2,480,705 (39.1)	2,362,865 (43.5)
Male	3,962,165 (62.5)	3,860,972 (60.9)	3,069,338 (56.5)
BMI			
<25	1,807,108 (28.5)	1,916,065 (30.2)	1,646,714 (30.3)
25-30	2,527,114 (39.9)	2,192,379 (34.6)	1,917,144 (35.3)
30-35	1,188,147 (18.8)	1,275,569 (20.1)	1,182,092 (21.8)
35-40	473,805 (7.5)	493,732 (7.8)	374,354 (6.9)
>40	339,093 (5.4)	463,932 (7.3)	311,899 (5.7)
Race			
White	5,820,237 (91.9)	5,695,610 (89.8)	4,920,104 (90.6)
Black	389,714 (6.2)	453,186 (7.1)	353,033 (6.5)
Asian	96,514 (1.5)	124,238 (2.0)	148,367 (2.7)
Other	28,802 (0.5)	68,643 (1.1)	10,699 (0.2)
Ethnicity			
Non-Hispanic	6,156,727 (97.2)	6,162,709 (97.2)	5,252,875 (96.7)
Hispanic	178,540 (2.8)	178,968 (2.8)	179,328 (3.3)
Smoking history			
Former smoker	3,214,373 (50.7)	2,987,986 (47.1)	2,662,133 (49.0)
Current smoker	3,120,894 (49.3)	3,353,691 (52.9)	2,770,070 (51.0)
Pack-years			
<40	1,887,954 (29.8)	1,953,135 (30.8)	1,750,120 (32.2)
41-50	1,825,926 (28.8)	2,077,722 (32.8)	1,744,923 (32.1)
51-60	702,183 (11.1)	885,207 (14.0)	706,200 (13.0)
61-70	412,970 (6.5)	400,932 (6.3)	354,027 (6.5)
71-80	476,849 (7.5)	321,865 (5.1)	218,038 (4.0)
81-90	414,873 (6.5)	247,792 (3.9)	289,209 (5.3)
90-100	242,812 (3.8)	162,741 (2.6)	174,804 (3.2)
>100	371,700 (5.9)	292,283 (4.6)	194,882 (3.6)
Comorbidities			
Asthma	648,243 (10.2)	835,553 (13.2)	661,766 (12.2)
Diabetes	1,057,324 (16.7)	1,331,638 (21.0)	1,027,559 (18.9)
Emphysema	672,675 (10.6)	648,028 (10.2)	688,122 (12.7)

© 2023 Robertson SE et al. *JAMA Network Open.*

Heart disease	1,110,125 (17.5)	1,250,381 (19.7)	888,138 (16.3)
Hypertension	3,169,649 (50.0)	3,441,984 (54.3)	2,743,312 (50.5)
Stroke	242,844 (3.8)	341,904 (5.4)	290,795 (5.4)
Education/marital status			
Marital status			
Never married	241,465 (3.8)	392,007 (6.2)	296,558 (5.5)
Married or living as married	4,201,070 (66.3)	4,106,128 (64.7)	3,234,968 (59.6)
Widowed	675,109 (10.7)	478,343 (7.5)	571,243 (10.5)
Separated	156,924 (2.5)	125,955 (2.0)	98,060 (1.8)
Divorced	1,060,699 (16.7)	1,239,244 (19.5)	1,231,374 (22.7)
Education			
Less than high school	1,318,086 (20.8)	1,163,258 (18.3)	912,074 (16.8)
High school graduate	2,414,799 (38.1)	2,400,685 (37.9)	1,916,866 (35.3)
Some college/AA degree	1,740,991 (27.5)	1,774,907 (28.0)	1,866,081 (34.4)
Bachelor's degree or above	861,391 (13.6)	1,002,827 (15.8)	737,182 (13.6)

© 2023 Robertson SE et al. *JAMA Network Open.*

eTable 10. Lung Cancer-Specific Mortality Results for the Transportability Analyses Using the Fully Adjusted Model, With Weights in the Transportability Analysis Trimmed to 99%

Analysis	Low-dose CT	Chest radiography	Rate ratio	Rate difference
NHIS 2005	310 (274, 351)	373 (334, 416)	0.83 (0.70, 0.98)	-63 (-119, -7)
NHIS 2010	325 (280, 376)	395 (347, 450)	0.82 (0.67, 1.00)	-71 (-141, 0)
Combined NHIS 2005 and 2010	313 (275, 355)	380 (275, 355)	0.82 (0.69, 0.98)	-67 (-126, -9)

© 2023 Robertson SE et al. *JAMA Network Open.*

eTable 11. All-Cause Mortality Results for Using the Fully Adjusted Model, With Weights in the Transportability Analysis Trimmed to 99%

Analysis	Low-dose CT	Chest radiography	Rate ratio	Rate difference
NHIS 2005	1480 (1404, 1560)	1565 (1488, 1647)	0.95 (0.88, 1.01)	-86 (-198, 26)
NHIS 2010	1638 (1541, 1741)	1691 (1597, 1791)	0.94 (0.87, 1.03)	-92 (-228, 44)
Combined NHIS 2005 and 2010	1522 (1442, 1607)	1615 (1532, 1702)	0.94 (0.87, 1.02)	-93 (-211, 25)

© 2023 Robertson SE et al. *JAMA Network Open.*

eFigure 2. Sensitivity Analysis Results, on the Rate Ratio Scale for Lung Cancer Mortality, for Violations of the Transportability Assumption

The black x corresponds to the base case of the main analysis, when the sensitivity parameters are set to 0. The estimated effect of screening (indicated by the color gradient levels) varies according to the value of the sensitivity parameters.

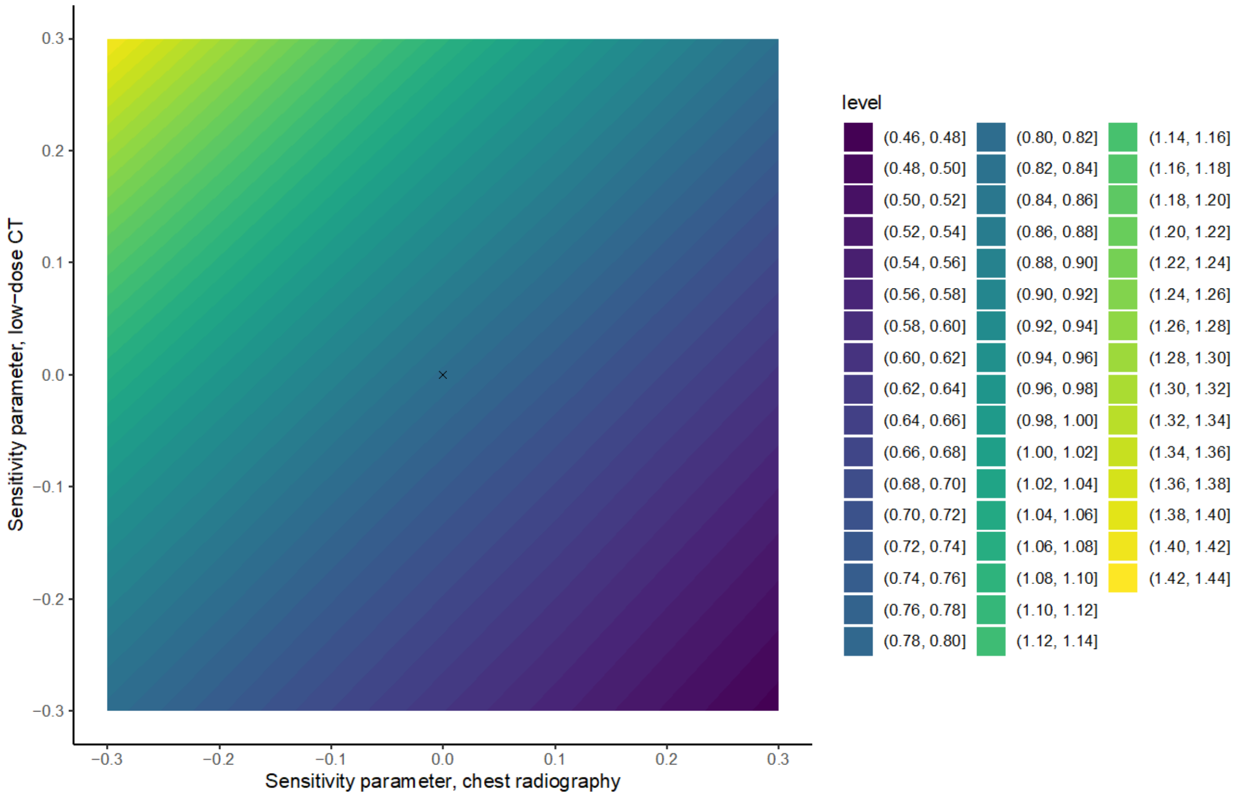

© 2023 Robertson SE et al. *JAMA Network Open.*

eReferences.

1. Dahabreh IJ, Robertson SE, Steingrimsson JA, Stuart EA, Hernán MA. Extending inferences from a trial to a new target population. Statistics in Medicine. 2020 Apr 6; PMID: 32253789

2. Li L, Shen C, Li X, Robins JM. On weighting approaches for missing data. Statistical Methods in Medical Research. 2013 Feb;22(1):14–30.

3. Scharfstein DO, McDermott A. Global sensitivity analysis of clinical trials with missing patient-reported outcomes. Statistical Methods in Medical Research. 2018 Mar 20;096228021875956.

4. Scharfstein D, McDermott A, Díaz I, Carone M, Lunardon N, Turkoz I. Global sensitivity analysis for repeated measures studies with informative drop-out: A semi-parametric approach. Biometrics. 2018 Mar;74(1):207–219.

5. Dahabreh IJ, Robins JM, Haneuse SJPA, Robertson SE, Steingrimsson JA, Hernán MA. Global sensitivity analysis for studies extending inferences from a randomized trial to a target population [Internet]. arXiv; 2022 [cited 2023 Oct 2]. Available from: http://arxiv.org/abs/2207.09982

6. Robins JM, Rotnitzky A, Scharfstein DO. Sensitivity analysis for selection bias and unmeasured confounding in missing data and causal inference models. Statistical models in epidemiology, the environment, and clinical trials. Springer; p. 1–94.

© 2023 Robertson SE et al. *JAMA Network Open.*
