# Supplementary material for: Comparing Lung Cancer Screening Strategies in a Nationally Representative US Population Using Transportability Methods for the National Lung Cancer Screening Trial
Source: JAMA Netw Open. 2024 Jan 30;7(1):e2346295. doi: 10.1001/jamanetworkopen.2023.46295 (PMC10828917; doi:10.1001/jamanetworkopen.2023.46295)
Supplement: Supplement 2. — Data Sharing Statement [file jamanetwopen-e2346295-s002.pdf]

Data Sharing Statement

Robertson. Comparing Lung Cancer Screening Strategies in a Nationally Representative US Population Using Transportability Methods for the National Lung Cancer Screening Trial. *JAMA Netw Open*. Published January 30, 2024. doi:10.1001/jamanetworkopen.2023.46295

Data
Data available: No

Additional Information
Explanation for why data not available: The data analyses in our paper used 2005 and 2010 NHIS data obtained from the IPUMS USA database. We also acknowledge the NCHS (National Center for Health Statistics) as the original data source and the U.S. Census Bureau. The data analyses used NLST data obtained from the National Cancer Institute (NCI). The content of this paper is solely the responsibility of the authors and does not necessarily represent the official views of AHRQ, PCORI, PCORI's Board of Governors, the PCORI Methodology Committee, NCHS, or NCI. The authors cannot share the data used in the analyses reported in this paper; the NHIS data are publicly available and the NLST data can be obtained following review by request to the NCI.
